# Teenage first-time mothers’ perceptions about their health care needs in the immediate and early postpartum period in Uganda

**DOI:** 10.1186/s12884-022-05062-7

**Published:** 2022-10-03

**Authors:** Mariam Namutebi, Dorcus Kabahinda, Scovia Nalugo Mbalinda, Racheal Nabunya, Dorothy Gingo Nanfuka, Lydia Kabiri, Tom Denis Ngabirano, Patience A. Muwanguzi

**Affiliations:** 1 Department of Nursing, College of Health Sciences, School of Health Sciences, Makerere University, Kampala, Uganda; 2 Department of Social Work and Social Administration, College of Humanities and Social Sciences, School of Social Sciences, Makerere University, Kampala, Uganda; 3 Department of Paediatrics and Child Health, College of Health Sciences, School of Medicine, Makerere University, Kampala, Uganda

**Keywords:** Teenage, First-time mothers, Postpartum, Health care needs, Perceptions

## Abstract

**Background:**

Teenagers have higher risks for complications during the intrapartum and postpartum periods. Although facility-based postpartum care focusses on preventing complications in mothers and babies, it is not understood what teenage-mothers’ perceptions are about their health care needs in the early postpartum period.

**Methods:**

An exploratory descriptive qualitative study was conducted in four health facilities in Uganda. In-depth interviews with 42 first-time teenage mothers aged 14 to 19 years were conducted between March and April 2020. Thematic analysis was done.

**Results:**

Two themes emerged, Health promotion and Rehabilitation and counseling. Teenage first time mothers desired to receive information about key issues like self and newborn care, breast feeding, immunization and family planning. They noted that health workers need to monitor their vital signs which aids in early diagnosis of complications, disease prevention/treatment of current conditions. Others felt that health workers are key in arbitrating between them and their estranged parents and also help to link them to community based organizations that can provide them with counseling and life skills.

**Conclusions:**

Teenage first-time mothers have many health care needs during the immediate and early postpartum period. This is a missed opportunity to provide health education and link them to sexual reproductive health services including family planning, breastfeeding clinics and other community based programs which provide life skills or continuing education for girls. Focusing on these needs and integration of services is key in providing holistic care to the teenagers. We propose that further research be done to explore how their health care needs change at 6 months post-delivery.

## Introduction and background

Globally, it is estimated that there are 12 million births among adolescents annually, and the adolescent birth rate remains the highest in the African continent [1, 2]. The teenage pregnancy rate in Uganda and the sub-Saharan region has remained high despite reported reductions over the decades [3–5]. The percentage of girls in Africa who have had a first birth before their 20th birthday is varied, with the lowest 31% in Ghana and the highest at 73% in Niger [5]. The birth rates in Uganda are varied by region and level of urbanization ranging from 24% in the central region to 31% in the eastern region [4].

Teenage first-time mothers face unique economic, social, and environmental challenges like family and peer isolation and stigma, inability to meet their financial, and the babies' needs [6–9]. Secondly, reports suggest that access to health care and health-seeking behavior among teenage mothers is lower than that of other mothers. Yet, they are at higher risks of developing complications and mortality during pregnancy and childbirth [7, 9]. Therefore, teenage first time mothers require more skilled attention during birth and in the postpartum period. Other studies have reported that teenagers and their babies are prone to illness due to their ill-preparedness for the challenges of motherhood, gaps in health information, and lack of support [10, 11]. Although the barriers to accessing maternity care among this population have been studied, there are few published studies that have explored the health care needs of the first-time teenage mothers during the postpartum period and how these can be met to reduce complications during this time period [6, 10, 12]. The current country postpartum care guidelines [13] are silent on the special care for teenage mothers although there is a need for healthcare workers to understand and integrate the health care needs for teenage mothers in their daily practice so as to improve client satisfaction, reduce complications and possibly increase the number of teenagers delivering at the health facilities. Therefore, this study explored the teenage first-time mothers’ perceptions of their health care needs during the immediate and early postpartum period.

## Methods

### The research team and reflexivity

MN, RN, SNM, TDN, and PAM are Nurse-Midwives; DGN is a Pediatrician while KD is a social worker. All the researchers and the research assistants had prior training and experience in conducting qualitative research. Daily reflective journals were kept by all the members collecting the data, and debrief meetings were held regularly by the two site teams. During the data collection process, MN, who was part of the training for the research assistants, gave each one a chance to share how each one perceived health care needs based on their backgrounds. The group then discussed how these perceptions could influence the data collection process and how to mitigate this. We had five members of the data collection team who had prior experience of postpartum care while the others had not interacted with any woman during this time period. It was agreed that those with prior experience would endeavor to keep our interviews focused on the research question and not ask leading questions or bias the respondents in light of our past experiences. Our previous thoughts about postpartum care were also noted and considered when the data analysis process begun.

#### Study population

The study participants were first-time teenage mothers aged 13 to 19 years receiving postpartum care or immunization services at the four selected health facilities in Soroti and Kalungu districts in Uganda. The Ugandan healthcare system is divided into several hierarchical levels of healthcare provision. At the highest level are the national specialized hospitals, followed by the national referral Hospitals, regional referral hospitals, district hospitals, health center IVs all the way down to the lowest rung of health center I level [14]. We conducted the study at two hospitals and two health center IVs from the two districts. The facilities at these levels were selected because, they can provide comprehensive emergency obstetric care. Secondly, because first-time, teenage mothers are at risk of complications, they are often referred to either a health center IV or hospital for their care. Hence, we expected to find our target population at the selected facilities. Soroti and Kalungu districts were selected because they both had high levels of teenage pregnancy according to the demographic health survey [4] . In Soroti district, we selected a public regional referral hospital and a private health centre IV, whereas, in Kalungu, we were at a public health centre and a private, not-for-profit Hospital.

#### Study design

We used an exploratory descriptive qualitative (EDQ) approach in this study because there was limited data about this critical phenomenon. This data was part of data collected for a phenomenological study that explored the lived experiences of first-time, teenage mothers receiving facility-based postpartum care. For this study, we explored their perceptions of their health care needs during this period. This study design was selected because it was appropriate to help us explore the thoughts, expectations, and perceived needs of the teenage postpartum mothers [15].

#### Sampling

The participants were purposively selected. This was done to ensure that the selected participants were of varied social demographic characteristics so as to ensure that the varied perceptions were captured if at all they existed.

#### Inclusion and exclusion criteria

All first-time, teenage mothers aged 13–19 years receiving postpartum care from the selected health facilities who; had delivered at a health facility or sought care soon after a home birth had come for either the 6 days or 6 weeks’ postpartum visit and were willing to participate in the study. We excluded all first-time, teenage mothers who could not speak any of the three languages (English, Luganda and Ateso) used in the study.

#### Data collection tool

Data were collected using an indepth interview guide in English and two other Ugandan languages, Ateso and Luganda. The guide was designed by the researchers from literature and translated to Luganda and Ateso by two language experts. The Luganda and Ateso interview guides were then back-translated to English to ensure consistency of the translation by different independent language experts. The guide contained two sections; social demographic characteristics and perceptions about the postpartum health care needs of first-time teenage mothers in the immediate and early postpartum period. Preliminary interviews were conducted among first-time teenage mothers purposively selected in Kalungu district. Changes to the tool were made based on the responses and suggestions from the first-time teenage mothers.

#### Data collection procedure

Data were collected by two of the researchers (MN and DK) and four research assistants. The team was comprised of three graduate nurses and three social workers. The researchers trained these in interviewing, communication skills, note-taking, and ethical conduct of research. Face to face In-depth interviews were conducted with 42 teenage mothers who met the eligibility criteria for inclusion in the study.

All teenage mothers attending the postnatal clinics in the selected facilities were provided with information about the study and informed consent sought from those who had come for their 6 days or 6 weeks’ postnatal visits. We approached 46 first-time teenage mothers who were waiting for postnatal care services at the health facilities over 5 weeks between March and April 2020. All of them consented to participate; however, four of them were unable to complete the interviews and requested to withdraw from the study due to emotional distress stirred by the questions. All the participants were interviewed in a secluded room identified at the facility (for privacy and to reduce interruptions during the interviews) after receiving their postpartum care. Permission was sought prior to audio recording the interviews. One research assistant was the interviewer while the other team member took notes. Each interview lasted between 30 minutes to 1 hour. Study participants’ enrollment continued until the point of saturation was reached (when no new data was being generated from the interviews). Data saturation was deemed to have been reached after 38 interviews, but the researchers conducted four more interviews after that to validate the data saturation. Participants, who experienced emotional distress during the study, were offered counseling services by the research assistants on the team that were social workers.

#### Data management and analysis

A clear file naming system was used. Data were tracked systematically and filed according to the participant’s number, facility, and district. Data were analyzed using the six steps of thematic data analysis including, familiarization with the data, code generation, searching for themes, reviewing the themes, defining and naming the themes, and report writing, as described by V Braun and V Clarke [16]. The audiotaped interviews were transcribed verbatim to ensure the preservation of the data. The transcripts were then translated to English by the research assistants who conducted the interviews. These transcripts were compared with the field notes written by the scribe for each interview to ensure consistency. The data were analyzed based on the definition of health care needs by Wright, Williams and Wilkinson [17], which is a gap in a person’s health state that would benefit from effective and appropriate care interventions or from wider social and environmental changes. Two researchers (MN and DK) read and re-read the transcripts and coded the data independently. They then reviewed them together with three research assistants to identify and highlight significant statements and quotes that provided an understanding of what the participants perceived to be their needs in the postpartum period and their expectations of the care. These significant statements were used to make clusters of meaning which helped the researchers identify codes, define subthemes and themes from the data. Agreement between the researchers and the research assistants on the meanings of the data and emerging themes was ensured through several online and physical meetings. These themes were then reviewed by the whole research team that refined and renamed them based on the components of health care needs by Wright, Williams and Wilkinson [17].

### Trustworthiness of data

Trustworthiness in qualitative research has been said to consist of four criteria, including; credibility, transferability, dependability, and confirmability [18]. To ensure credibility in this study, the research team collected the data over a 6 week period from mid-March to April 2020. In that time, they were also able to informally observe the process of providing care to the clients at the health facilities. Three researchers (MN and KD) coded the data separately for dependability, and the team discussed the codes they each developed and agreed on the final codes. The procedures followed in this study have been written clearly to ensure that they can be repeated in other contexts.

## Results

We interviewed 42 first-time, teenage mothers, 16 from Kalungu district and 26 from Soroti district. Their level of education ranged from primary four to senior six; 25 had primary level education while 17 had gone to secondary school. There were two participants who were still students, while another two had undertaken an apprenticeship in tailoring. The rest of the social demographic information is in Table 1.

**Table 1:** Social demographic characteristics of the respondents

Variable	Frequency (percentage
District	
Soroti	26 (61.9)
Kalungu	16 (38.1)
Age of respondent in years	
14	1 (2.4)
16	4 (9.5)
17	5 (11.9)
18	12 (28.6)
19	20 (47.6)
Level of education	
Primary	25 (59.5)
Secondary	17 (40.5)
Occupation	
None	19 (45.2)
Tailor	2 (4.8)
Farmer	11 (26.2)
Student	2(4.8)
others	8 (19.0)

### Health care needs of first-time, teenage mothers

**Fig. 1:**
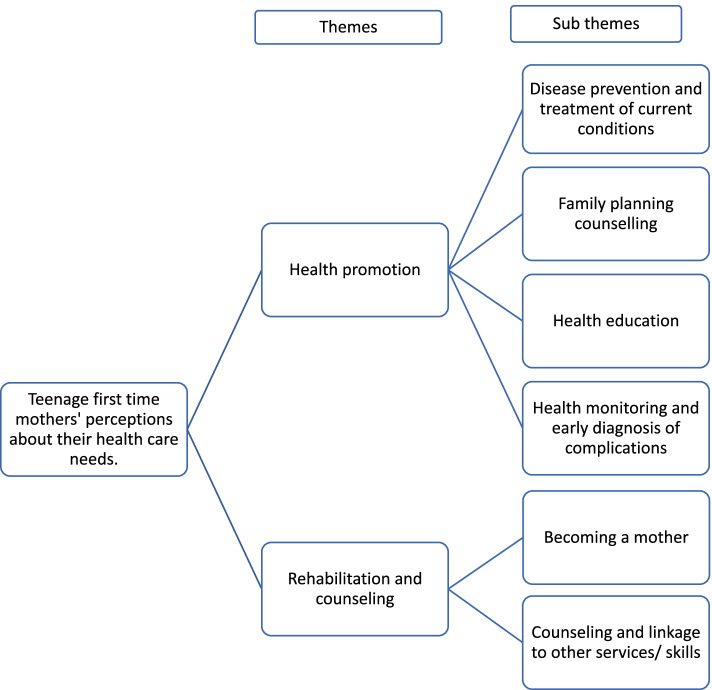
Thematic presentation of the findings

The health care needs identified in this study were subdivided into two main themes (see Fig. 1): health promotion, and Rehabilitation and counseling. In the paragraphs that follow, the findings are presented in the form of texts and narrative quotes from the participants arranged within the subthemes and themes identified.

### Health promotion

Under the theme of health promotion, three sub-themes were identified, including health education, health monitoring and diagnosis of complications, disease prevention, and treatment of complications.

#### Health education

Many first-time mothers recognized that they initially had little to no information about caring for themselves and their babies after delivery. Those that were taught were glad and appreciated the information given however those that didn’t receive postpartum information felt that they could have benefited from it.

*The nurse didn’t tell me anything concerning my hygiene and that of the baby … hmmmm just imagine nothing like how to clean where the baby tore me, not even how to breastfeed the baby, all they said after putting me on bed was don’t bath the baby till tomorrow, I was not told why they said so and also what kind of water and the time to bath (shaking the head in form of regret) Participant 7, 18 years).*

*I thought since this is my first child, where I take my antenatal care, they should be teaching me how to carry a baby, how to breastfeed a child, all those things I did not see those things, just to get medicine, check you and then and go. When I got here, I did not know how to carry a child to breastfeed, I got to know all that from here, and I was asked if I was not taught, I told them “No.”* (Participant 27, 18 years).

However, there was a general perception that although information was given about self-care and care for the baby, it was not always adequate, and thus, some participants were not sure of what and how to do what they were instructed to do. This led to mixed feelings and even frustration among the participants as they navigated life after discharge from the hospital.

*Yes, mostly I wish the nurse told me how to take care of my wound [episiotomy site] and what is required. I felt terrible pain when at home, like after sitting. I felt a lot of pain, yet I was not told what to do about it* [face changed, became moody hand around her stomach. Wiped tears from her eyes and looked at her child]*.* (Participant 3, 19 years).

#### Health monitoring and early diagnosis of complications

Following delivery, the first-time teenage mothers expected to be constantly checked on with their babies. They felt that the constant monitoring was a sign that the midwife cared and reassured them that all was well with them and their babies. Failure on the midwives’ part to check on the clients greatly reduced their satisfaction with the care. The quote that follows is from a 19-year-old teenage first-time mother who delivered before arriving at the health facility,

*My expectation was that the midwife would give me so much care to make sure that I got all the care needed to keep my baby and I alive. I did not expect to give birth and be abandoned by the health worker. I expected her to check on me, take my pulse, see if I am doing well, just like she did.* (Participant 40, 19 years).

Another 19-year-old teenage first-time mother from Kalungu articulated the need for constant monitoring of postpartum clients stating that the monitoring should be the same for all clients to ensure those with complications are recognized quickly. Her sentiments are verbalized in the following quote,

*By constantly monitoring the patients, the health workers can know who may have issues. Sometimes health workers may not pay a lot of attention to people who delivered naturally without complications. If everyone is given the same kind of attention, then those who develop complications after birth would be diagnosed and treated quickly …*. (Participant 36, 18 years).

#### Disease prevention and treatment of current conditions

When complications arose, there was an expectation that the health workers would be able to assess, diagnose, and treat the condition of the mother or the baby before they were discharged from the hospital. One participant, whose baby was readmitted to the hospital after discharge, explains,

*The baby had ‘kamunye’[jaundice] and cried all night. She did not want to breastfeed no matter how hard I tried. I felt bad about it. I really wished the midwife had helped; the baby started crying while we were in the hospital, and we tried to get the midwife to help, but she would just say breastfeed the baby unless you want us to fix a tube* [nasogastric tube] *into her to feed by it.* (Participant 35, 19 years).

Another participant whose baby was admitted for breathing problems also narrated,

*They took a long time to remove the things they take out of the nose, and because of this delay, the child could not breathe. (*Participant 28, 19 years).

Another aspect of disease prevention that the first-time, teenage mothers highlighted was through ensuring health facility and bathroom/ toilet hygiene. This was recognized as a measure that could reduce the risk for infections among the mothers and their babies.

*The environment was dirty, the toilets were dirty, the bathrooms were dirty, and if one needed to use a bathroom, one had to clean it themselves to the point that one could pick infections.* (Participant 34, 17 years).

#### Family planning counseling

Although some participants did not think that they needed information about family planning immediately after delivery, others were concerned that while they had been told to ensure that they had a long birth to birth interval they were not told how to achieve that. One 19-year-old teenage mother remarked,

*The midwife did not talk to me about family planning. But I do not think that a midwife should tell me about family planning immediately after birth. I think that happens later (*Participant 34).

Another participant shared,

*Yes, the midwife checked my BP, pulse; she also checked my blood levels and told me that they were low.* [She gestured, opening her eye] *She did not check the uterine contraction, and she did not talk to me about family planning. (*Participant 35 19 years).

### Rehabilitation and counseling

This theme had three sub-themes, becoming a mother, counseling, and linkage to care.

#### Becoming a mother

The delivery of the baby was recognized by both the midwives and first-time teenage mothers as an entry into a new role. Some of the mothers reported being congratulated by the health workers upon becoming a mother. Several teenage first-time mothers admitted ignorance concerning their new roles as mothers and would have appreciated someone telling them what the new phase of life had in store for them and how to cope with the new challenges. One 18 year old housewife further explains;

*“What I would have wanted to know, is how you handle a child, how to care for a child. I think once you have given birth, you no longer have an option, you have to know”* (Participant 26 18 years).

Another participant decried the lack of preparation below,

*A hospital with a good standard the nurses/midwives are supposed to care for us, handle us very well, instead of them shouting at you telling you that why get pregnant or give birth before time, they handle us very well, then sometimes they need to tell you what’s it is that is happening to you and even what is in it you have just entered [the responsibilities attached] and many times girls go there instead of telling them, they just abuse them. (Participant 25, 18 years).*

In instances where the first-time, teenage mothers felt ill-prepared when complications arose, they were blamed on the midwives; sometimes, they were even suspected of intentionally providing no information.

*Why couldn’t they tell me that breastfeeding hurts at the beginning, or do they require me to pay money for that also? Imagine not giving me the necessary information I needed about taking care of the wound and enough medication, and on reaching home, I stayed for a period of less than a week, and the wound started hurting like I was being stitched afresh. I could not sit, not even telling me what to do hmmm* [wiped tears from her eyes and went silent for about 5 minutes] *I hate everything.*

Some first-time mothers sought advice from older women who sometimes gave information based on the cultural traditions or practices, which could have been based on misconceptions about the cause of ailments. This is illustrated by an excerpt from one participant who was convinced that her baby had tummy trouble, probably colic, because the midwives did not massage her stomach while at the hospital.

*I felt bad for sure, because how and why didn't they tell me how to massage the baby’s stomach, if not for the old women who came around to check on me and helped me to keep massaging the baby’s stomach till it went back to normal* (Put on a sad face, went silent for 5mins) (Participant 7, 19 years).

#### Family counseling and linkage to other services

One of the participants’ needs was for an arbitrator to mediate between them and their estranged families who would advocate for reconciliation and convince their parents to send them back to school. Though the participants knew that the midwives might not be able to solve the issues, they felt that the midwives would help to link them to organizations that could help. An example of such mitigation attempts is shown below.

*The health workers, while at the hospital before discharge, organized a counseling session for my mother and me. They asked her to forgive me and consider a second chance saying that it was still possible for me to get back to school and continue with my education. But my parents did not consider that option, and that is where my education ended*. **[**At this point in the interview, she was beginning to lose the sense of calm she had before. Her voice became shaky. The tempo and speed at which she spoke became slower. She bowed her head, and tears began to fall profusely. By now, she was barely speaking in a whisper; there was a shaking in the sound of her voice. I could see a lot of unresolved pain and a sense of regret. Her look was distant as though away from where we were seated. She kept muttering to herself] *“nandisomye” I should have been allowed to study!* (Participant 37, 18 years).

## Discussion

Two themes emerged from this study and these were health promotion and Rehabilitation and counseling. Participants in this study were aged 14–19 years and had limited experience with care after delivery. As such, many of them expressed a desire to receive more information about self-care, new born care, family planning and when to return to hospital during the discharge time. This is in line with findings from studies done among adult first time mothers. At most facilities in Uganda, group antenatal health education is conducted each morning before the provision of care starts, there are also sessions to educate postpartum mothers about what to do after discharge at most units.

Our study highlights the need to emphasize the health education needs reported by these mothers during these sessions. It is also possible that the health workers are often overwhelmed by the numbers of patients hence are unable to take off time to provide the necessary guidance. This may due to low staff to patient ratios that have been noted in other studies done in low income settings [19] and the observations of some of the participants in this study which makes it hard for the health worker to provide the recommended care as per the national guidelines. This finding is similar to women in a study by P Larkin, CM Begley and D Devane [20] who recognized that the midwives were often quite busy and unable to provide this information. One of them suggested employment of people to just provide this information to them if the midwives are not able. This is similar to findings from studies done among first-time adult mothers, which indicated that new ways of information provision and alterations in the content of antenatal education sessions may be needed to incorporate post-delivery care needs and coping strategies and that busyness hindered communication [21–23].

The participants also desired consistent monitoring during the postpartum period to detect and prevent maternal and new-born complications. Participants were afraid that selective monitoring of only women deemed at risk could result in missing any others who developed complications later, hence cost the life of the mother or the baby. This desire may be driven from stories they come across, given that Uganda still has a high maternal mortality ratio standing at 336 per 100,000 live births [4]. To some of the participants, the constant monitoring was a sign that the health worker cared about their wellbeing. Participants expressed similar concerns in other studies, like the need for assurance of health or a return to health for the mother and the baby during the early postpartum period [24].

Mothers are often concerned and distraught when they or their baby develops complications after delivery. In this study, participants who developed complications or whose babies required more medical attention noted that having keen midwives or better care from the health workers would avail timely diagnosis and treatment of such conditions. For those participants, this caused stress and fear as they faced the uncertainty of the possible outcomes. A systematic review of what matters to women in the postpartum period found that having optimum health for the mother and baby was very important, which may explain the findings in this study [24].

The mothers felt that they ought to be congratulated and adequately prepared for their new roles as mothers. They also needed affirmation and counsel on how to become good mothers. According to RT Mercer [25], women experience different phases as they adapt themselves to the new role of motherhood. They need affirmation from others to build self-confidence in their performance of the role. This affirmation can be sought from the health workers, significant others, and those who have been mothers before. This highlights the need to provide information and practical help with mothering roles for first-time mothers to reduce their anxiety over the roles since they tend to seek advice from lay people who may not give accurate explanations [10, 25, 26].

There was controversy over the need for family planning information immediately after delivery in this study with some desiring to be given information about the services while others thought that should be delayed. This may be because the participants were young, having their first baby, and some were not married or were living with their parents. The other reason could be that the mothers were facing very many challenges with their new status as a mother that the issue of family planning counseling could wait. On the other hand, some participants felt they needed to be given information in order to make decisions about their fertility. A study done in Uganda reported that teenagers expected to receive family planning education at the health facility. The difference may be because they recruited teenage mothers who were not in the postpartum period [12]. This study highlights the fact that this is a missed opportunity to provide health information and sexual reproductive health services to these clients.

In this study, some of the participants were still students or desired to return to school in due course. Together with the low social-economic status, they had experienced already; some participants suggested that linkages to organizations that can help them gain occupational skills and family counseling sessions be integrated into the care to persuade their parents to take them back to school after the delivery. A clinical trial done in the united states [27] found that integrating life skills in the care improved maternal self-esteem and parenting skills among teenage mothers. In our study, though this was outside the clinical roles of the midwives, the participants having experienced animosity and tension at home, were reaching for any hope of relief and chance for a better future. Health workers among teenage mothers with HIV have also suggested offering them and their babies linkage to HIV care [11, 28]. Integration of counseling and rehabilitation services in the general maternity care can thus be considered as an approach to improving teenagers’ lives in general [28].

During the data collection process, many of the participants were overwhelmed and emotional at the memory of their experiences during the postpartum period. Some even chose to withdraw from the study. This was significant because it showed how having unmet care needs affected the psychological wellbeing of these participants. These findings concur with those from a Belgian study where women felt they needed psychological support during this time. It is therefore important that counselors and health care providers recognize the effect of these experiences on the teenagers' mental health, and engage them to enable them share their experiences, and what they have been facing during this time in order to improve their wellbeing [29, 30]. The use of pregnancy support groups in aiding teenagers to cope with pregnancy and postpartum challenges has also been suggested previously by a study done in our setting [31].

We also noted the need to provide holistic care to these mothers since the participants we interviewed in our study were not just distressed about their current state of motherhood but also pined after lost opportunities, strained relationships with parents and what the future held for them and their babies. Health workers need to consider the psychosocial, developmental as well as the physiological status of their clients while providing care because all these dimensions do affect how the clients perceive the care provided. This may be considered a universal need for all clients but because teenagers often experience stress and stigma when they get pregnant, health workers need to pay close attention to their psychological health [29, 30].

### Study strengths and limitations

The main strength of this study was the qualitative methodology used in data collection and triangulation of coders during data analysis to ensure credibility of our data. Data were collected from two districts in Uganda which ensured regional variation among participants, however it may not be representative of teenage first time mothers in the whole country.

## Conclusions

Teenage first-time mothers have many health care needs during the immediate and early postpartum period. Therefore, we recommend that; health education should be done throughout pregnancy and the early postpartum period about motherhood, self-care and new-born care. Secondly, this is an opportunity to provide or link the first-time teenage mothers to sexual reproductive health services including Family planning and the breastfeeding clinics. Thirdly, health facilities may need to identify and partner with reputable community based programs to provide life skills or continuing education for girls who are unable to continue with school. The Ministry of health's care guidelines need to be updated to capture the health care needs of this special population and all health care providers trained in identifying and providing care to teenage first time mothers especially in areas where the teenage pregnancies are high. Lastly, the provision of adequate and quality care based on these identified needs could improve the patients’ experience and satisfaction with postpartum care. This coupled with integration of services is also key in meeting the needs of the teenagers and providing holistic care. We also propose that further research be done on the health care needs of this population between 6 months to 1 year post-delivery so to as to explore how their needs change over time.

## Data Availability

The datasets used and/or analyzed during the current study are available from the corresponding author on reasonable request**.**

## Abbreviations

HIV: Human Immunodeficiency Virus
WHO: World Health Organization
ANC: Antenatal care
EDQ: Exploratory descriptive qualitative study
